# Beat-to-beat blood pressure variability, hippocampal atrophy, and memory impairment in older adults

**DOI:** 10.1007/s11357-024-01303-z

**Published:** 2024-08-05

**Authors:** Trevor Lohman, Isabel Sible, Allison C. Engstrom, Arunima Kapoor, Fatemah Shenasa, Elizabeth Head, Lorena Sordo, John Paul M. Alitin, Aimee Gaubert, Amy Nguyen, Kathleen E. Rodgers, David Bradford, Daniel A. Nation

**Affiliations:** 1 Leonard Davis School of Gerontology, University of Southern California, Los Angeles, CA USA; 2 Department of Psychology, University of Southern California, Los Angeles, CA USA; 3 Department of Psychological Science, University of California, Irvine, Irvine, CA USA; 4 Department of Pathology and Laboratory Medicine, University of California, Irvine, Irvine, CA USA; 5 Center for Innovations in Brain Science, Department of Pharmacology, University of Arizona, Tucson, AZ USA; 6 Keck School of Medicine, University of Southern California, Los Angeles, CA USA

**Keywords:** Blood pressure variability, Hippocampus, Glial fibrillary acidic protein, Plasma neurofilament light, Memory impairment

## Abstract

Visit-to-visit blood pressure variability (BPV) predicts age-related hippocampal atrophy, neurodegeneration, and memory decline in older adults. Beat-to-beat BPV may represent a more reliable and efficient tool for prospective risk assessment, but it is unknown whether beat-to-beat BPV is similarly associated with hippocampal neurodegeneration, or with plasma markers of neuroaxonal/neuroglial injury. Independently living older adults without a history of dementia, stroke, or other major neurological disorders were recruited from the community (*N* = 104; age = 69.5 ± 6.7 (range 55–89); 63% female). Participants underwent continuous blood pressure monitoring, brain MRI, venipuncture, and cognitive testing over two visits. Hippocampal volumes, plasma neurofilament light, and glial fibrillary acidic protein levels were assessed. Beat-to-beat BPV was quantified as systolic blood pressure average real variability during 7-min of supine continuous blood pressure monitoring. The cross-sectional relationship between beat-to-beat BPV and hippocampal volumes, cognitive domain measures, and plasma biomarkers was assessed using multiple linear regression with adjustment for demographic covariates, vascular risk factors, and average systolic blood pressure. Elevated beat-to-beat BPV was associated with decreased left hippocampal volume (*P* = .008), increased plasma concentration of glial fibrillary acidic protein (*P* = .006), and decreased memory composite score (*P* = .02), independent of age, sex, average systolic blood pressure, total intracranial volume, and vascular risk factor burden. In summary, beat-to-beat BPV is independently associated with decreased left hippocampal volume, increased neuroglial injury, and worse memory ability. Findings are consistent with prior studies examining visit-to-visit BPV and suggest beat-to-beat BPV may be a useful marker of hemodynamic brain injury in older adults.

**Supplementary Information:**

The online version contains supplementary material available at 10.1007/s11357-024-01303-z.

## Introduction

Increased visit-to-visit blood pressure variability (BPV) is an age-related hemodynamic risk factor for dementia [1], and is predictive of decreased hippocampal and medial temporal lobe (MTL) volume [2, 3], cognitive decline [4], and neurodegeneration [5]. The relationship between BPV and neurodegeneration may be related to increased risk for cerebrovascular lesion burden [6], or decreased cerebral perfusion [7].

Previous research shows that visit-to-visit blood pressure variability is influenced by medication adherence [8]. Beat-to-beat BPV may therefore represent a more reliable tool for prospective risk assessment, however, no studies to date have examined beat-to-beat BPV in relation to markers of neurodegeneration, such as hippocampal atrophy or plasma markers of neuroaxonal/neuroglial injury and neurodegeneration.

Two plasma biomarkers [9], neurofilament light (NfL) and glial fibrillary acidic protein (GFAP), have emerged as sensitive markers of neuroaxonal [10] and neuroglial injury [11] respectively. Both markers are associated with aging and cognitive ability [12, 13], with GFAP elevation sensitive to even subtle central nervous system injury [11] and NfL sensitive to neuroaxonal injury and cerebral small vessel disease [14, 15]. Plasma GFAP is also a sensitive marker of astrogliosis [16] that is associated with neurodegeneration [17, 18], while plasma NfL is a neurodegenerative susceptibility marker that is predictive of future abnormal morphological changes in the brain [19]. No study to date has assessed whether beat-to-beat BPV is associated with these plasma biomarkers, despite the established relationship between visit-to-visit BPV and cerebrovascular [20, 21] and neurodegenerative disease [3].

If beat-to-beat BPV is associated with hippocampal atrophy, cognitive impairment might also be expected, particularly within the memory domain [22]. The present study examines the relationship between elevated beat-to-beat BPV and hippocampal volumes, plasma NfL and GFAP levels, and memory ability.

## Methods

### Participants

Participants were recruited from Orange County communities through outreach events, mailing lists, word-of-mouth, online portals, a research volunteer registry, and through the University of California Irvine (UCI) Alzheimer’s Disease Research Center (ADRC), and all procedures were conducted as part of the Vascular Senescence and Cognition (VaSC) Study at UCI. Older adults aged 55 to 89 years who were living independently were included (Table 1). Study exclusions were a prior diagnosis of dementia, history of clinical stroke, family history of dominantly inherited neurodegenerative disorders, current neurological or major psychiatric disorders that may impact cognitive function, history of moderate-to-severe traumatic brain injury, current use of medications impairing the central nervous system, current organ failure or other uncontrolled systemic illness, and contraindications for brain MRI. Eligibility for the study was verified by a structured clinical health interview and review of current medications with the participant and, when available, a knowledgeable informant study partner. All participants underwent neurological and neuropsychological evaluations performed using the Uniform Data Set (UDS), and additional neuropsychological tests, as described in the neuropsychological testing section. This study was approved by the UCI Institutional Review Board, and all participants gave informed consent. The anonymous data that support the findings of this study are available upon reasonable request from the corresponding author, DAN, through appropriate data-sharing protocols.

**Table 1:** Participant characteristics and demographics

Variable name	Overall (*n* = 104) Mean ± SD	Biomarker Subset (*n* = 56) Mean ± SD	*P*
Age	69.5 ± 6.7 (55–89)	69.8 ± 7.3 (55–89)	.81^a^
Sex (*n*, female %)	65 (62.5)	35 (62.5)	> .99^b^
Vascular risk factors			
Hypertension (*n*, %)	37 (35.6)	18 (32.1)	.79^b^
High Cholesterol (*n*, %)	51 (49.0)	28 (50.0)	.91^b^
Diabetes (*n*, %)	11 (10.6)	5 (8.9)	.74^b^
Current smoker (*n*, %)	33 (31.7)	19 (33.9)	.78^b^
Cardiovascular disease (*n*, %)	11 (10.6)	6 (10.7)	.98^b^
Atrial fibrillation (*n*, %)	5 (4.8)	3 (5.4)	.88^b^
Transient ischemic attack (*n*, %)	2 (1.9)	0 (0)	.30^b^
*APOE* genotype			
* APOE2* carriers (*n*, 2/3%)	3 (2.9)	3 (5.4)	.43^b^
* APOE2* carriers (*n*, 2/4%)	0 (0)	0 (0)	
* APOE3* homozygotes (*n*, 3/3%)	45 (43.3)	26 (46.4)	.70^b^
* APOE4* carriers (*n*, 3/4)	42 (40.4)	26 (46.4)	.54^b^
* APOE4* homozygotes	2 (1.9)	1 (1.8)	.95^b^
Unknown or missing	12 (11.5)	0 (0)	
Left hippocampal volume (mm^3^)	3811.52 ± 464.01	3802.03 ± 512.74	.91^a^
Right hippocampal volume (mm^3^)	3975.82 ± 413.04	3959.66 ± 454.87	.83^a^
Plasma GFAP (pg/ml)	147.81 ± 74.47	147.81 ± 74.47	
Plasma NfL (pg/ml)	18.84 ± 8.39	18.84 ± 8.39	
Memory composite *z*-score	.57 ± .82	.59 ± .86	.89^a^
Story memory delayed recall *z*-score	.47 ± 1.07	.45 ± 1.11	.94^a^
Word list delayed recall *z*-score	.90 ± 1.20	.84 ± 1.33	.76^a^
Word list delayed recognition *z*-score	.25 ± .94	.21 ± .94	.80^a^
Attention/executive function composite *z*-score	− .12 ± .58	− .10 ± .57	.87^a^
Trail-making test A *z*-score	− .08 ± .77	− .16 ± .76	.52^a^
Trail-making test B *z*-score	.04 ± .65	.03 ± .67	.95^a^
Stroop color and word test *z*-score	− .30 ± .84	− .15 ± .84	.27^a^
Language composite *z*-score	.06 ± .76	− .009 ± .81	.59^a^
Categorical verbal fluency *z*-score	− .33 ± .87	− .37 ± .85	.77^a^
Confrontational naming *z*-score	− .08 ± 1.20	− .24 ± 1.43	.47^a^
Phonemic verbal fluency *z*-score	.58 ± .83	.58 ± .77	.98^a^
BPV (mmHg)	1.69 ± .79	1.73 ± .81	.74^a^
Average SBP (mmHg)	133.01 ± 17.03	131.83 ± 17.22	.68^a^

*SD* standard deviation, *APOE* apolipoprotein e, *GFAP* glial fibrillary acidic protein, *NfL* neurofilament light, *BPV* blood pressure variability indexed as systolic blood pressure average real variability, *SBP* systolic blood pressure, *A* two-sample* t*-test, *B* Pearson’s chi-squared test

### Continuous BP data acquisition

Participants were asked to take medications as normally prescribed and abstain from caffeine on the morning of data collection. Beat-to-beat BP measurements were obtained continuously during supine rest in a 3 T Siemens MRI scanner, using an MRI-compatible non-invasive continuous BP finger cuff device (Biopac®). First, the participant rests for 3 min in the supine position prior to the calibration period. During calibration, BP waveforms are acquired by the continuous monitoring device and 2 static pressures are simultaneously acquired using a calibrated, MRI-compatible automatic BP device with an inflatable brachial artery cuff (TeslaDUO). These static pressures are used to calibrate the continuous BP monitor using the Caretaker® system (Biopac®). After calibration, continuous BP was monitored during MRI for 7 min and further data processing was performed as previously described [23].

### Systolic blood pressure average real variability

Beat-to-beat BPV is quantified as systolic blood pressure (SBP) average real variability (ARV), a measure of systolic beat-to-beat BPV which has been demonstrated as reliable in older adults [23, 24] regardless of antihypertensive medication use [23]. ARV calculates the average of absolute changes between consecutive blood pressure readings and is calculated as:

$$\text{ARV}=\frac{1}{n-1} \sum_{k=1}^{n-1}|{BP}_{k+1}-{BP}_{k}|$$

where n represents the number of blood pressure readings obtained during continuous blood pressure monitoring and k represents the beat index of the readings as previously described [23, 25]. Systolic ARV was chosen as the measure of beat-to-beat BPV in the present study due to its increased reliability compared to other BPV measures such as standard deviation and coefficient of variation [23], and its decreased susceptibility to outliers [24]. Additionally, ARV has the advantage of considering the temporal ordering of systolic BP measurements and is therefore a more specific measure of beat-to-beat fluctuations in blood pressure [23, 26].

### Vascular risk factors (VRF)

Vascular risk factor (VRF) burden was determined through clinical interviews with the participant and informant (when available), and review of current medications and medical history. The assessed VRFs included a history of cardiovascular disease (e.g., heart failure, angina, stent placement, coronary artery bypass graft, intermittent claudication), hypertension, hyperlipidemia, type 2 diabetes, atrial fibrillation, left ventricular hypertrophy, and transient ischemic attack. Total VRFs were summed for each participant and elevated VRF burden was defined as ≥ 2 VRFs (vs. 0–1) as described previously [27, 28].

### APOE genotyping

Fasted blood samples were obtained by venipuncture and used to determine the participant’s *APOE* genotype. Genomic DNA was extracted using the PureLink Genomic DNA Mini Kit (Thermo). The isolated DNA concentration was determined using a NanoDrop One (Thermo). DNA was then stored at − 80 °C for long-term storage. Isolated DNA was first diluted to a concentration of 10 mg/μL. PCR reactions were performed in a final volume of 25 μL containing 25 ng DNA, 0.5 μM of both forward and reverse primers (forward: ACGGCTGTCCAAGGAGCTG; reverse: CCCCGGCCTGGTACACTG), and 1 × SYBR Green Master Mix (Qiagen) diluted in H2O. For the amplification, a T100 Thermal Cycler (BioRad) was used with the following settings: 95 °C for 10 min; 32 cycles of 94 °C for 20 s, 64 °C for 20 s, and 72 °C for 40 s; followed by 72 °C for 3 min. Fifteen microliters of the DNA PCR product was digested with Hhal-fast enzyme at 37 °C for 15 min. The digested PRC product was added to a 3% agarose gel in 1 × borax buffer for gel electrophoresis. The gel was run at 175 V for 25 min and visualized on ChemiDoc (BioRad) with a GelRed 10,000 × gel dye. *APOE4* carrier status was defined as *APOE4* carriers (at least one copy of the ε4 allele) or *APOE4* non-carriers (no copies of the ε4 allele), as previously described [29]. All analyses were performed at the same lab at the University of Arizona (KER).

### Brain volumes

All participants underwent brain MRI scans conducted on a 3 T Siemens Prisma scanner with 20-channel head coil. High-resolution 3D T1-weighted anatomical (Scan parameters: TR = 2300 ms; TE = 2.98 ms; TI = 900 ms; flip angle = 9 deg; FOV = 256 mm; resolution = 1.0 × 1.0 × 1.2 mm^3^; Scan time = 9 min) images were acquired, using 3-dimensional magnetization-prepared rapid gradient-echo (MPRAGE) sequences.

For region-of-interest (ROI) analysis, post-processing of T1 scans was accomplished in FreeSurfer 7.4.1 [30] using an automated segmentation algorithm that is robust to anatomical variability including ventricular enlargement associated with neurological diseases and aging for quantification of bilateral hippocampal volumes [31]. After automated segmentation, each individual subject was checked for any inaccuracies or misclassifications; manual corrections were made as needed with FreeSurfer’s built-in editing tools, cases were then re-processed, and resulting volumes were used for analyses.

### Plasma biomarkers

Blood plasma from fasted blood samples was separated by centrifugation and stored at -80 °C until AD biomarker assays. All plasma Aβ_40_ and Aβ_42_ concentrations were obtained using the digital immunoassay, Simoa Neurology 3-Plex A (N3PA) Advantage Kit (Quanterix). Plasma total tau was also obtained but not analyzed due to questions regarding its relationship with brain AD pathological changes [32]. Plasma levels of GFAP and NfL were determined using a single molecule array, (Simoa®) Neurology 2-Plex B (N2PB) Kit (Quanterix), following the manufacturer’s protocol on the HD-X machine. Accepted ranges were as follows: NfL = 0– ~ 2000 pg/mL and GFAP = 0– ~ 40,000 pg/mL. All biomarker assays were conducted in the same lab at UCI (EH).

### Neuropsychological testing

All participants underwent a clinical interview and comprehensive neuropsychological assessment by a trained technician or doctoral student under the supervision of a licensed clinical neuropsychologist. The assessment included multiple tests of memory, attention/executive function, and language. All neuropsychological testing and diagnostic assessments were conducted blinded to all clinical, biomarker, and imaging findings. Composite scores were created for memory, attention/executive function, and language. A memory composite score was created by averaging the demographically corrected (age, sex, and education) z-scores from three memory tests which included story memory delayed recall (either Weschler Memory Scale–Revised [WMS-R] Logical Memory-II [33] or Craft story delayed recall [34, 35]), word list delayed recall (Rey Auditory Verbal Learning Test [RAVLT] Trial 7 [36] or CERAD word list 30-min delayed recall [37]), and word list delayed recognition (RAVLT Recognition [36] or CERAD word list 30-min delayed recognition [37]). An attention/executive function composite was created by averaging the demographically corrected *z*-scores from the attention/executive function tests which included the trail-making test A [38], trail-making test B [38], and one other attention/executive test which included either the Golden Stroop color and word test [39], the D-KEFS color-word interference test [40], or the digit span backward test. Lastly, a language composite score was created by averaging the demographically corrected z-scores of the three language tests which included semantic verbal fluency (Animals) [41], confrontational naming (Boston Naming Test [42] or multilingual naming test [43]), and phonemic verbal fluency (FAS) [41].

### Data analysis

One hundred five participants underwent continuous BP monitoring and brain MRI. One participant was excluded after 3 SD outlier screens (+ 4.35 SD left hippocampal volume, + 4.31 SD right hippocampal volume) resulting in a total analyzed sample size of 104 for volumetric analyses. A subset of these participants also had neuropsychological testing characterization (*n* = 103) available for analysis. Demographically adjusted neuropsychological component scores were screened for outliers and averaged into memory (*n* = 93), attention/executive (*n* = 101), and language (*n* = 101) composite scores as previously described. The relationship between beat-to-beat BPV and demographically adjusted neuropsychological domain composite scores was investigated. A subset of 56 participants with plasma GFAP and NfL assays were also analyzed.

Linear regression models assessed whether beat-to-beat BPV was associated with hippocampal atrophy, neuropsychological domain composite *z*-scores, and plasma markers of neuroaxonal/neuroglial injury with and without adjustment for age and sex where applicable (volumetric and plasma biomarker analyses), and for average beat-to-beat SBP and VRF burden. Volumetric analyses also included total intracranial volume (TIV) as a covariate. All analyses were performed in R [44].

Additional sensitivity analyses were performed for the significant age, sex, VRF burden, and average beat-to-beat SBP-adjusted findings. These analyses included additional correction for *APOE4* carrier status, plasma Aβ_42/40_, and where appropriate, neuropsychological testing site. Benjamini–Hochberg false discovery rate (FDR) correction [45] was also applied for the primary analyses (L/R hippocampal volumes, plasma GFAP, plasma NfL, and memory composite *z*-score).

## Results

Participant demographics and characteristics for the total analyzed sample and the plasma biomarker subset are displayed in Table 1 with between-group statistical comparisons. No significant differences in participant characteristics were observed between the overall sample and biomarker subset.

**Fig. 1:**
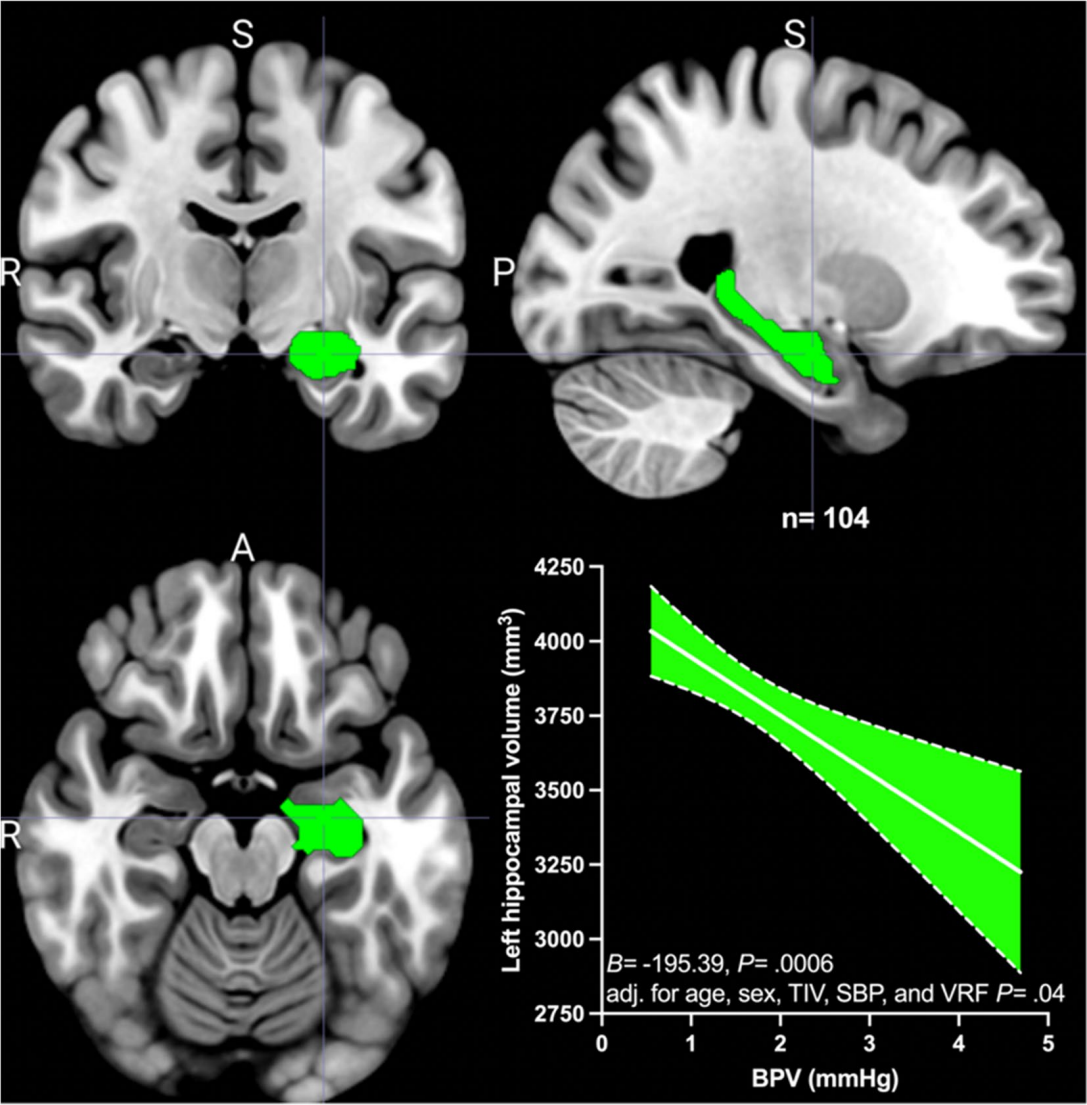
The relationship between blood pressure variability (BPV) and left hippocampal volume. BPV is measured as systolic blood pressure (SBP) average real variability. The left hippocampus region of interest is shown in green in coronal, sagittal, and transverse sections. Unstandardized beta (*B*) and *p* value (*P*) are shown for the univariate analysis, as is the *p* value for age, sex, total intracranial volume (TIV), SBP, and vascular risk factor burden (VRF) adjusted multiple linear regression analysis

Increased beat-to-beat BPV was significantly associated with decreased left hippocampal volume (*B* =  − 195.39, *P* = 0.0006), and remained so after adjustment for age, sex, TIV, average SBP, and VRF burden (*B* =  − 111.40, *P* = 0.04) (Fig. 1). BPV was also associated with decreased right hippocampal volume (*B* =  − 139.86, *P* = 0.0006; however, this relationship was attenuated by age, sex, TIV, average SBP, and VRF burden adjustment (*B* =  − 74.70, *P* = 0.10) .

**Fig. 2:**
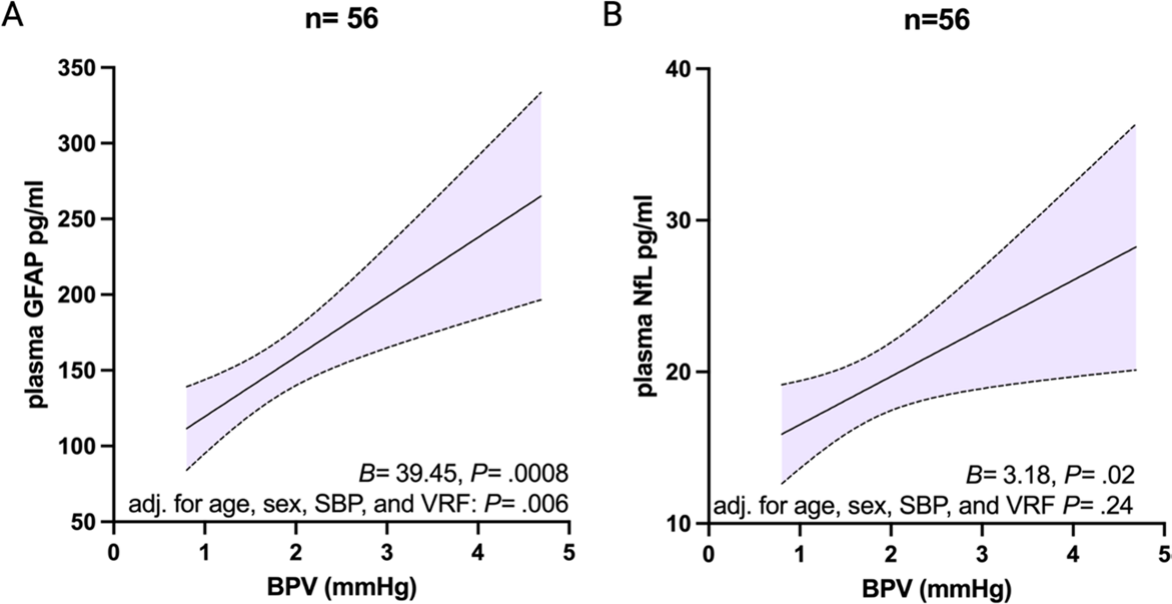
The relationship between blood pressure variability (BPV) and **A** plasma glial fibrillary acidic protein (GFAP) pg/ml and **B** plasma neurofilament light (NFL) pg/ml. BPV is measured as systolic blood pressure (SBP) average real variability. Unstandardized beta (*B*) and *p* value (*P*) are shown for the univariate analysis, as is the *p* value for age, sex, SBP, and vascular risk factor burden (VRF) adjusted multiple linear regression analyses

BPV was significantly associated with plasma GFAP (*B* = 39.45, *P* = 0.0008), and remained so after age, sex, average SBP, and VRF burden adjustment (*B* = 29.70, *P* = 0.006) (Fig. 2a). SBP ARV was also associated with plasma NfL (*B* = 3.18, *P* = 0.02), but this relationship was attenuated by age, sex, average SBP, and VRF burden adjustment (*B* = 1.58, *P* = 0.24) (Fig. 2b).

**Fig. 3:**
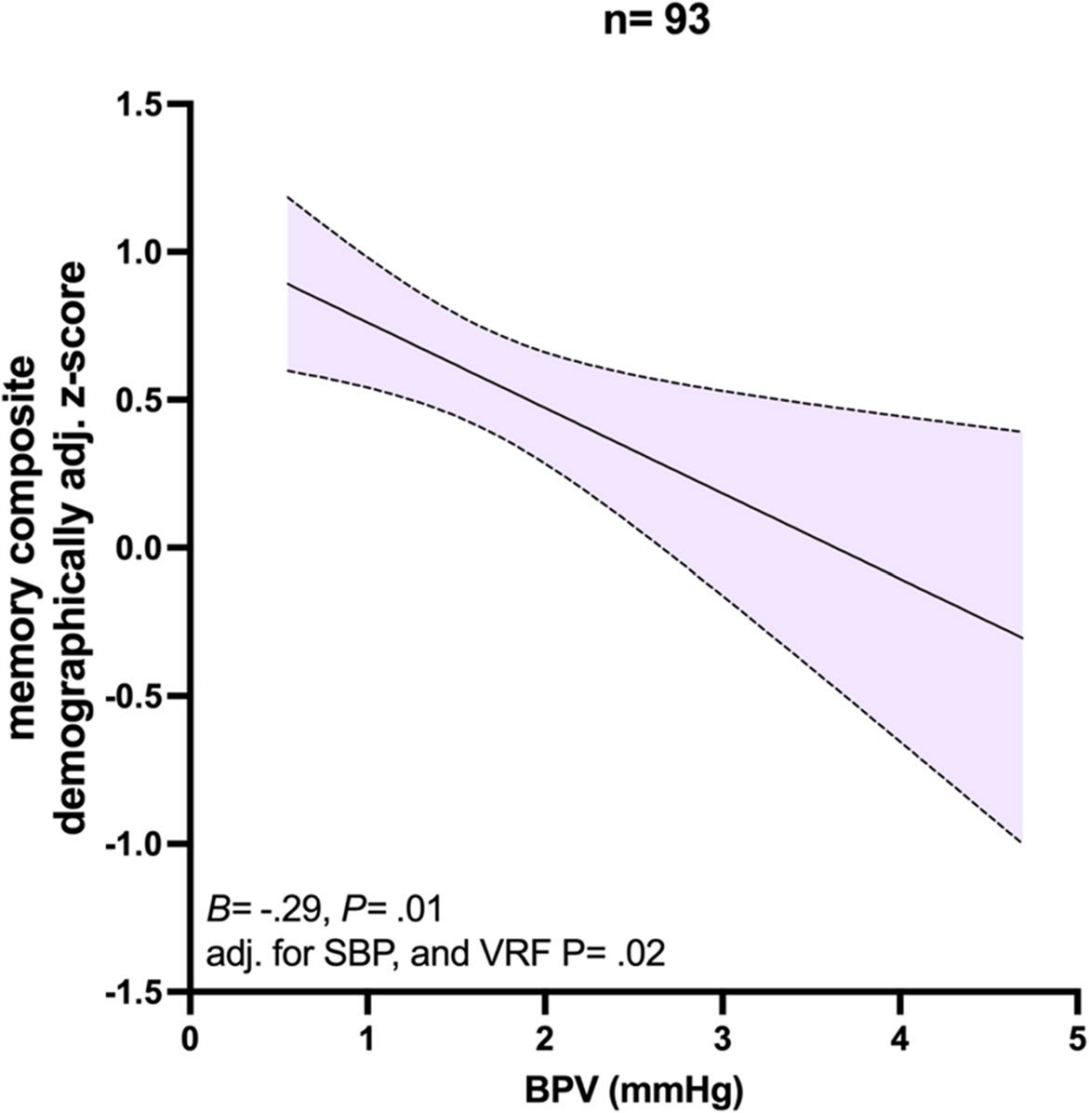
The relationship between blood pressure variability (BPV) and demographically adjusted memory composite score. The relationship between BPV, measured as systolic blood pressure (SBP) average real variability, and demographically adjusted (age, sex, and years of education) memory composite *z*-score. Unstandardized beta (*B*) and *p* value (*P*) are shown for univariate analysis, as is the *p* value for the SBP and vascular risk factor burden (VRF) adjusted multiple linear regression analysis

BPV was significantly associated with demographically adjusted (age, sex, and education) memory composite *z*-score in both the univariate analysis (*B* =  − 0.29, *P* = 0.01) and adjusted for SBP and VRF burden as shown in Fig. 3. No significant relationships existed between BPV and attention/executive function composite *z*-score (*B* = 0.03, *P* = 0.70) or language composite *z*-score (*B* =  − 0.02, *P* = 0.82).

### Sensitivity analyses and multiple comparisons

All age, sex, VRF, TIV (where applicable), and average beat-to-beat SBP-adjusted significant BPV findings remained significant after additional sensitivity analysis which included *APOE4* carrier status, plasma Aβ_42/40_ (Supplementary Table S1–S2), and where applicable, neuropsychological testing site (Supplementary Table S3) as covariates. Additionally, FDR correction was applied to the primary univariate analyses including L/R hippocampal volumes, plasma GFAP, plasma NfL, and memory composite score. All significant univariate findings survived FDR correction (L/R hippocampal volume, plasma GFAP, plasma NfL, and memory composite *z*-score).

## Discussion

The present study finds that elevated beat-to-beat BPV is significantly associated with decreased left hippocampal volume and worse memory ability in independently living older adults. BPV was also associated with plasma GFAP, a marker of neuroinflammation, neurodegeneration, and reactive astrogliosis that is associated with cortical volume loss, and cognitive decline [18, 46]. The observed relationships were independent of age, sex, SBP, and VRF adjustment. These results are consistent with prior studies of visit-to-visit BPV predicting hippocampal atrophy and memory decline but extend these findings to the more reliable [23, 24] beat-to-beat ARV measure of blood pressure fluctuation. This is also the first study to examine BPV levels related to the plasma GFAP marker of neuroglial injury.

Delayed free recall is associated with MTL atrophy, specifically within the hippocampus [47–50], consistent with the well-established role of hippocampal atrophy in age-related episodic memory decline [51]. The finding that BPV was related to left hippocampal atrophy adds convergent validity to the memory impairment observed in the present study. This effect is further confirmed by the observation that plasma GFAP, a sensitive marker of central nervous system injury, reactive astrogliosis, neuroinflammation, and neurodegeneration [11, 12, 16, 18] was also related to elevated beat-to-beat BPV.

Although plasma GFAP displays a strong correlation with cerebral amyloidosis [16], it is not a specific marker of Alzheimer’s disease pathology and shows diagnostic accuracy in differentiating between healthy controls and individuals with frontotemporal dementia, progressive supranuclear palsy, corticobasal syndrome, Lewy bodies dementia, and cognitively impaired individuals with suspected non-AD pathophysiology [52]. Thus, GFAP represents a sensitive marker of astrogliosis [53] and neuroinflammation [11] associated with numerous neurodegenerative pathologies [11]. The observed association between elevated beat-to-beat BPV and GFAP suggests that BPV may be related to glial injury and neuroinflammation, or it could be related to neural injury more broadly, with future studies needed to elucidate these specific mechanistic relationships.

The hippocampus is selectively vulnerable to age-related vascular pathology [54], chronic hypoperfusion [55], and hypoxic injury [56]. Increased BPV predicts a future longitudinal decline in cerebral perfusion [57], even when average pressure is held within a narrow range as part of a clinical trial [7]. Since elevated BPV is associated with decreased whole-brain cerebrovascular reactivity [58], increased cerebrovascular lesion burden [20], and beat-to-beat BPV is associated with decreased hippocampal perfusion specifically [59], it is possible that the hippocampus would display susceptibility to hemodynamic risk factors like elevated beat-to-beat BPV. In the present study, BPV was associated with left but not right-sided hippocampal atrophy, which is consistent with previous research showing a left-sided neurodegenerative vulnerability [60], and left-sided cerebrovascular disease susceptibility [61], potentially related to increased left-sided hemodynamic stress [61]. The left-sided effect seen in the present study then is also consistent with the susceptibility of the hippocampus to vascular insult.

The present study adds the novel findings that elevated beat-to-beat BPV is associated with left hippocampal atrophy, memory decline, and plasma GFAP in a cognitively healthy sample while controlling for plasma Aβ_42/40_ and Alzheimer’s disease genetic risk factors. This suggests that elevated beat-to-beat BPV may be an early biomarker of hippocampal atrophy and episodic memory decline in cognitively healthy older adults, independent of Alzheimer’s disease-specific pathophysiological changes. Previous research has shown that the adverse effect of elevated visit-to-visit BPV on cognitive decline and neurodegeneration is increased in *APOE4* carriers [3, 4]. Future investigations in larger samples should assess whether the effect of beat-to-beat BPV is also moderated by *APOE4* carrier status.

Strengths of the present study include the convergent findings in older adults showing elevated BPV in relation to three interconnected markers of neurodegeneration, including neuroimaging volumetrics of the hippocampus, a plasma biomarker of neurodegeneration, and episodic memory impairment. Other strengths include the analysis of beat-to-beat BPV in a well-characterized sample, including a participant subset with plasma biomarkers of neuroaxonal and neuroglial injury. Study limitations include not all participants having plasma biomarker data available for analysis and the exclusion of participants with a history of stroke which limits generalizability. Also, causal inference is limited due to the cross-sectional study design. Neurodegenerative diseases are associated with autonomic dysfunction [62], and since neurodegeneration can occur within central autonomic brain regions [63], the possibility for reverse causation exists [64]. However, current evidence supports blood pressure variation being predictive of future neurocognitive decline [1], and studies that have assessed for reverse causation in related disorders such as cerebral small vessel disease have failed to support the reverse causation hypothesis [21].

The present study findings indicate further research is needed into beat-to-beat BPV as a potentially modifiable risk factor for neurodegeneration and neurocognitive decline with major clinical implications for older adults at risk for dementia.

## Supplementary Information

Below is the link to the electronic supplementary material.

Supplementary file1 (DOCX 16 KB)

## Data Availability

The anonymous data that support the findings of this study are available upon reasonable request from the corresponding author, DAN, through appropriate data-sharing protocols.

## Abbreviations

ARV: Average real variability
BPV: Blood pressure variability
GFAP: Glial fibrillary acidic protein
NfL: Neurofilament light
MTL: Medial temporal lobe
SBP: Systolic blood pressure
