# Supplementary material for: Beat-to-beat blood pressure variability, hippocampal atrophy, and memory impairment in older adults
Source: GeroScience. 2024 Aug 5;47(1):993–1003. doi: 10.1007/s11357-024-01303-z (PMC11872826; doi:10.1007/s11357-024-01303-z)
Supplement: Supplementary file 1 — Supplementary file1 (DOCX 16 KB) [file 11357_2024_1303_MOESM1_ESM.docx]

**Supplementary Table S1: Unstandardized regression parameters for the relationship between beat-to-beat BPV and left hippocampal volume adjusted for age, sex, TIV, VRF, average SBP, APOE4 carrier status, and plasma Aβ_42/40_.**

| Variable name | *B* | SE | *P* |
| --- | --- | --- | --- |
| Beat-to-beat BPV (mmHg) | -109.20 | 50.46 | .03 |
| Age (years) | -25.41 | 6.47 | .0002 |
| Sex (male) | 267.70 | 105.10 | .01 |
| TIV (mm^3^) | .00085 | .0003 | .004 |
| ≥2 VRF | -86.41 | 76.73 | .26 |
| Average SBP (mmHg) | -1.09 | 2.39 | .65 |
| *APOE4* carrier | -27.35 | 78.91 | .73 |
| Plasma Aβ_42/40_ | 6381.00 | 5177.00 | .22 |

**Dependent variable: Left hippocampal volume (mm^3^).** BPV: blood pressure variability indexed as systolic blood pressure (SBP) average real variability, TIV: total intracranial volume, VRF: vascular risk factor burden, *APOE4:* apolipoprotein e4.

**Supplementary Table S2: Unstandardized regression parameters for the relationship between beat-to-beat BPV and plasma GFAP adjusted for age, sex, TIV, VRF, average SBP, APOE4 carrier status, and plasma Aβ_42/40_.**

| Variable name | *B* | SE | *P* |
| --- | --- | --- | --- |
| Beat-to-beat BPV (mmHg) | 32.82 | 9.96 | .002 |
| Age (years) | 2.84 | 1.25 | .03 |
| Sex (male) | -53.66 | 14.76 | .0007 |
| ≥2 VRF | -1.66 | 14.81 | .91 |
| Average SBP (mmHg) | -.33 | .47 | .48 |
| *APOE4* carrier | 12.81 | 15.28 | .41 |
| Plasma Aβ_42/40_ | -2469.20 | 972.07 | .01 |

**Dependent variable: plasma glial fibrillary acidic protein (GFAP)** BPV: blood pressure variability indexed as systolic blood pressure (SBP) average real variability, VRF: vascular risk factor burden, *APOE4:* apolipoprotein e4.

**Supplementary Table S3: Unstandardized regression parameters for the relationship between beat-to-beat BPV and demographically adjusted memory composite z-score adjusted for VRF, average SBP, APOE4 carrier status, neuropsychological testing site, and plasma Aβ_42/40_.**

| Variable name | *B* | SE | *P* |
| --- | --- | --- | --- |
| Beat-to-beat BPV (mmHg) | -.29 | .12 | .02 |
| ≥2 VRF | -.34 | .19 | .07 |
| Average SBP (mmHg) | .003 | .006 | .57 |
| *APOE4* carrier | .31 | .19 | .10 |
| Neuropsychological testing site | .32 | .15 | .04 |
| Plasma Aβ_42/40_ | 1.16 | 11.84 | .92 |

**Dependent variable: demographically (age, sex, education) adjusted memory composite z-score.** BPV: blood pressure variability indexed as systolic blood pressure (SBP) average real variability, VRF: vascular risk factor burden, *APOE4:* apolipoprotein e4.
